# MicroRNA-197 regulates chondrocyte proliferation, migration, and inflammation in pathogenesis of osteoarthritis by targeting EIF4G2

**DOI:** 10.1042/BSR20192095

**Published:** 2020-09-16

**Authors:** Shijie Gao, Liang Liu, Shibo Zhu, Dawei Wang, Qiang Wu, Guangzhi Ning, Shiqing Feng

**Affiliations:** International Science and Technology Cooperation Base of Spinal Cord Injury, Tianjin Key Laboratory of Spine and Spinal Cord Injury, Department of Orthopedics, Tianjin Medical University General Hospital, Tianjin 300052, China

**Keywords:** EIF4G2, inflammation, miR-197, osteoarthritis, treatment

## Abstract

Recent studies have demonstrated that microRNAs (miRNAs) are involved in many pathological conditions including osteoarthritis (OA). In the present study, we aimed to investigate the role of miR-197 in OA and the potential molecular mechanism. The expression levels of miR-197 were detected by quantitative real-time PCR analysis. Cell proliferation and migration abilities were performed by 3-(4,5-dimethylthiazol-2-yl)-2,5-di-phenyltetrazolium bromide and transwell assays. The concentrations of inflammatory cytokines, including IL-1β, IL-6, and TNF-α, were detect using ELISA assay. Furthermore, luciferase reporter and rescue assays were applied to identify the functional target gene of miR-197 in OA. The results showed that miR-197 expression was significantly down-regulated in the OA cartilage tissues compared with normal cartilage tissues, accompanied by up-regulation of EIF4G2 expression. An inverse correlation was found between EIF4G2 and miR-197 expressions in OA cartilage tissues. Treatment with miR-197 mimics promoted the growth and migration abilities of chondrocytes, while miR-197 inhibitors induced the opposite effects. Furthermore, restoration of miR-197 significantly decreased IL-1β, IL-6, and TNF-α expression, whereas knockdown of miR-197 led to a induction in these inflammatory mediators. Moreover, EIF4G2 was predicted and confirmed as a directly target of miR-197. Overexpressed miR-197 could down-regulate EIF4G2 expression in chondrocytes, while miR-197 knockdown could elevate EIF4G2 expression. Additionally, EIF4G2 overexpression reversed the effects of miR-197 mimics on chondrocytes proliferation, migration, and inflammation. Taken together, our study demonstrated that miR-197 promotes chondrocyte proliferation, increases migration, and inhibits inflammation in the pathogenesis of OA by targeting EIF4G2, indicating the potential therapeutic targets of the miR-197/EIF4G2 axis for OA treatment.

## Introduction

Osteoarthritis (OA) is a common chronic osteochondral unit disease that involves not only cartilage but also meniscus, synovial membrane, subchondral bone, and infrapatellar fat pad [1,2]. OA usually slowly progressive, and accompanies cartilage degeneration with a loss of joint space that causes pain and disability [3]. OA is a leading cause of physical disability, pain, and shortening of working life in middle-aged and older people worldwide [4]. Multiple factors are involved in the etiology of OA, including aging, body weight, mechanical stress, trauma, and systemic related inflammatory diseases [5]. It has been reported that immune and inflammatory responses both play important roles in the pathological progress of OA [6,7]. Currently, the pain control and symptom management are the two primary treatment goals for OA, but the high incidence of OA places a significant economic burden on whole society [8]. Until now, the accurately mechanisms underlying the complex pathogenesis of OA are still not fully clarified. Therefore, it is necessary to explore the new regulatory mechanism underlying OA pathogenesis and develop novel targeted approaches for OA treatment.

A growing body of evidences have suggest that chondrocytes are the key cells in articular cartilage tissues and play an important role in the pathological progression of OA [9]. Moreover, multiple studies have found that dysregulated expression of some genes in chondrocytes are associated with the proliferation and inflammation responses, and that these genes are indispensable in cartilage homeostasis [10]. MicroRNAs (miRNAs) are non-protein coding RNA transcripts around 22 nucleotides in length [11]. They play key roles in the epigenetics for human diseases, thereby regulating target gene expression and downstream pathways [12]. Increasing studies have demonstrated that miRNAs play key roles in a variety of biological processes, such as cell metabolism, growth, differentiation, inflammation, and migration [13]. Importantly, dysregulation of miRNAs was discovered in the progression of OA, and could serve as new biomarkers for OA diagnosis and treatment [14,15]. For example, the dysregulation of miR-140-3p and miR-140-5p in synovial fluid correlates with the severity of OA [16]. miR-218-5p is a novel inducer of cartilage destruction via modulation of PI3K/Akt/mTOR signaling, inhibition of endogenous miR-218-5p expression/activity appears to be an attractive approach to OA treatment [17]. Recent study has described that miR-15a-5p promotes the degeneration of chondrocytes by targeting parathyroid hormone-related protein and may be a potential biomarker of OA [18].

miRNA-197 (miR-197) is located in human chromosome 1p13.3, and is reported to participate in the regulation of various cellular biological processes [19]. Ni et al. [20] suggested that miR-197 acts as a prognostic marker and inhibits cell invasion in hepatocellular carcinoma. Zhang et al. [21] demonstrated that miR-197 induces epithelial–mesenchymal transition and invasion through the down-regulation of HIPK2 in lung adenocarcinoma. Moreover, miR-197 predicts anti-inflammatory response to TNF-α inhibitor etanercept in rheumatoid arthritis [22]. miR-197 levels decrease as the severity of liver disease symptoms became aggravated in hepatitis B virus (HBV)-infected patients, and exogenous expression of miR-197 represses IL-18 expression at both the mRNA and protein levels in THP-1 cells [23]. However, the function and potential molecular mechanism of miR-197 involved in OA pathogenesis remain unknown. In the present study, we intended to investigate the effects of miR-197 overexpression and knockdown on the proliferation, migration, and inflammation abilities of chondrocytes, as well as the functional mechanism of miR-197 impacting OA. Our findings suggested that the miR-197/EIF4G2 axis functions as important roles in OA pathogenesis.

## Materials and methods

### Patients and specimens

Forty-one OA articular cartilage tissues were obtained from OA patients who underwent total knee arthroplasty, including 25 male and 16 female, with age range from 42 to 67 years. Twenty-nine normal articular cartilage tissues were obtained from trauma patients without OA history who underwent knee cartilage repair surgery, including 19 male and 10 female, with age range from 34 to 72 years. The cartilage tissues were immediately frozen in liquid nitrogen after the surgical resection and further stored at −80°C. Written informed consent was also obtained by all of the patients. The present study has been adhered to the standards of the Ethics Committee on Human Experimentation at Tianjin Medical University General Hospital and conducted in accordance with the regulations of the Helsinki Declaration (2000).

### Primary chondrocyte isolation and culture

The human OA cartilage tissues were treated and digested with 0.2% collagenase II (Thermo Fisher Scientific, Waltham, MA, U.S.A.), and primary chondrocytes were obtained as described previously [24]. The cells were cultured in the Dulbecco’s modified Eagle’s medium (DMEM; Invitrogen, Carlsbad, CA, U.S.A.) containing 15% fetal bovine serum (FBS; Invitrogen, Carlsbad, CA, U.S.A.), 100 units/ml of penicillin, and 100 units/ml of streptomycin for 24 h at 37°C with 95% air. Subsequently, the chondrocytes were filtered through a 0.075 mm cell strainer (Sigma, St Louis, MO, U.S.A.) and washed with sterile phosphate-buffered saline (PBS). After 3 weeks, chondrocytes at the second passage were obtained for further experiments.

### RNA extraction and quantitative real-time PCR analysis

Total RNA was isolated from cartilage tissues and chondrocytes by using TRIzol reagent (Invitrogen, Carlsbad, CA, U.S.A.), according to the manufacturer’s information. Each RNA samples were reverse transcribed into cDNA with the Mir-X™ miRNA First-Strand Synthesis Kit (Clontech, Mountain View, CA, U.S.A.) with 1 μg of RNA. Quantitative real-time PCR was conducted with the Mir-X™ miRNA qRT-PCR TB Green™ Kit (Clontech) on the ABI7500 Sequence Detection System (Thermo Fisher Scientific, Waltham, MA, U.S.A.). RNU6B and GAPDH were used as endogenous controls of miR-197 and EIF4G2, respectively. The primer sequences were showing as follows: EIF4G2, 5′-GGGTCATACTGCTGATTGTGGA-3′ (forward) and 5′-GAATGTGGTGCTTTGCTTCTGT-3′ (reverse); GAPDH, 5′-GGAGCGAGATCCCTCCAAAAT-3′ (forward) and 5′-GGCTGTTGTCATACTTCTCATGG-3′ (reverse); miR-197, 5′-ATTACTTTGCCCATATTCATTTTGA-3′ (forward) and 5′-ATTCTAGAGGCCGAGGCGGCCGACATGT-3′ (reverse); RNU6B, 5′-CTCGCTTCGGCAGCACA-3′ (forward) and 5′-AACGCTTCACGAATTTGCGT-3′ (reverse). The PCR reaction protocols were set 10 min at 95°C, followed by 40 cycles of 20 s at 95°C, 30 s at 55°C and 30 s at 72°C. The miR-197 and EIF4G2 expression results were presented as fold changes relative to RNU6B and GAPDH and were calculated by the 2 ^–ΔΔCT^ method [25]. The experiments were run in triplicate.

### Transfection

The miR-197 mimics, mimics control, miR-197 inhibitors, and inhibitors control were synthesized and obtained from RiboBio Co., Ltd. (Guangzhou, China) to overexpressing and knockdown the expression levels of miR-197. The pcDNA3.1-EIF4G2 vector and control vector were purchased at Genechem Co., Ltd. (Shanghai, China) to up-regulate the expression of EIF4G2. Chondrocytes were cultured in six-well plates with appropriate cells for 24 h at 37°C with 95% air. All oligonucleotides and/or plasmids were transfected into chondrocytes by using Lipofectamine® 3000 Transfection Reagent (Invitrogen, Carlsbad, CA, U.S.A.) according to the manufacturer’s information. Twenty-four hours after transfection, the cells were collected for subsequent experiments.

### 3-(4,5-dimethylthiazol-2-yl)-2,5-di-phenyltetrazolium bromide (MTT) assay

The chondrocytes were seeded into 96-well plates with a density of 5 × 10^3^ cells per well at different time points after transfection (0, 24, 48 and 72 h). Subsequently, 15 μl of 0.5% 3-(4,5-dimethylthiazol-2-yl)-2,5-di-phenyltetrazolium bromide (MTT) reagent (Sigma, St Louis, MO, U.S.A.) was added into each well for incubation 4 h at 37°C. Then the supernatant was removed and 120 μl of dimethylsulfoxide (Sigma) was added to dissolve the formazan precipitates for incubation another 4 h at 37°C. Optical densities were measured at 450 nm of each well with a Versamax microplate reader (Molecular Devices, Sunnyvale, CA, U.S.A.). The results were obtained from three independent experiments in triplicate.

### Transwell assay

The migration ability of chondrocytes was assessed by transwell assay with a 8-μm pore size polycarbonate membrane filter (BD Biosciences, Bedford, MA, U.S.A.). A totol of 3 × 10^5^ chondrocytes were placed in the upper chamber with 200 μl of DMEM, and the lower chamber was filled with 500 μl of DMEM medium containing 15% FBS. After incubation for 24 h in a 37°C with 95% air, the cells remaining on the upper surface of the membrane were removed with cotton swabs, while the migrated cells on the lower surface were fixed with 75% methanol for 30 min and then stained with 0.5% Crystal Violet solution for 20 min at 37°C. The migrated cells were photographed and counted by using an inverted fluorescence microscope (BZ 9000; Keyence Co., Osaka, Japan). The experiments were run in triplicate.

### Enzyme–linked immunosorbent assay (ELISA) assay

To determine the concentrations of inflammatory cytokines, the levels of IL-1β, TNF-α, and IL-6 in chondrocytes transfected with oligonucleotides and/or plasmids were detected. The transfected cells were cultured in six-well plates for 48 h. The chondrocytes were lysed and the supernatant was collected, and analyzed using the Human IL-1β, TNF–α, and IL-6 ELISA Kit (Beyotime Biotechnology, Shanghai, China), according to the manufacturer’s protocols. The experiments were performed in triplicate and repeated three times.

### Western blotting

The transfected chondrocytes were washed with cold PBS and lysed in RIPA reagent (Invitrogen, Carlsbad, CA, U.S.A.). The proteins were quantified using the BCA Protein Assay kit (Pierce Biotechnology, Rockford, IL, U.S.A.) and electrophoresed on a 10% SDS-PAGE gel (Beyotime Biotechnology). Then, the proteins were transferred to polyvinylidene fluoride membranes (Millipore, Bedford, MA, U.S.A.), and blocked with 5% nonfat milk for 2 h at 37°C. The membranes were incubated with primary antibodies against EIF4G2 (Santa Cruz Biotechnology, Santa Cruz, CA, U.S.A.) and GPAHD (Santa Cruz Biotechnology) at 4°C overnight. All primary antibodies were diluted in 1% bovine serum albumin (Beyotime Biotechnology) solution with a dilution of 1:2000. After the membranes were washed with TBST buffer, and incubated with HRP-conjugated secondary antibody (Santa Cruz Biotechnology). Signals of bands were recorded using ECL detection system (Pierce Biotechnology) according to manufacturer’s information. Data were analyzed using Quantity One software (Bio-Rad, Hercules, CA, U.S.A.).

### Luciferase reporter assay

The amplified 3′-UTR sequences of EIF4G2 were cloned to the pmirGLO luciferase vector (Promega, Madison, WI, U.S.A.) to build the wild-type (Wt) EIF4G2 luciferase vector, while the mutated 3′-UTR sequences of EIF4G2 were cloned to the pmirGLO luciferase vector to form the mutant (Mut) EIF4G2 luciferase vector. For luciferase reporter assay, the transfected chondrocytes were plated in 24-well plates with a density of 6 × 10^4^ cells per well, and cultured with Wt or Mut EIF4G2 luciferase vector for 48 h. Lysates were collected, and luciferase activities were consecutively measured by the dual-luciferase reporter system (Promega). Firefly luciferase activity was normalized against renilla luciferase activity. The experiments were performed in triplicate and repeated three times.

### Statistical analysis

The SPSS 22.0 software (SPSS, Inc., Chicago, IL, U.S.A.) was used for statistical analysis. All data were presented as the means ± standard deviation (SD) from three independent experiments in triplicate. Two group comparisons were performed with two-sided Student’s *t*-test. Multiple group comparisons were analyzed with two-sided one-way ANOVA and Tukey’s post hoc test. The normality of the data distribution was evaluated by the Kolmogorov–Smirnov test, and the correlations between EIF4G2 and miR-197 expression were determined by Spearman’s correlation analysis. A value of *P* less than 0.05 was considered to statistically significant.

## Results

### Expression of miR-197 and EIF4G2 in human OA cartilage tissues

**Figure 1:**
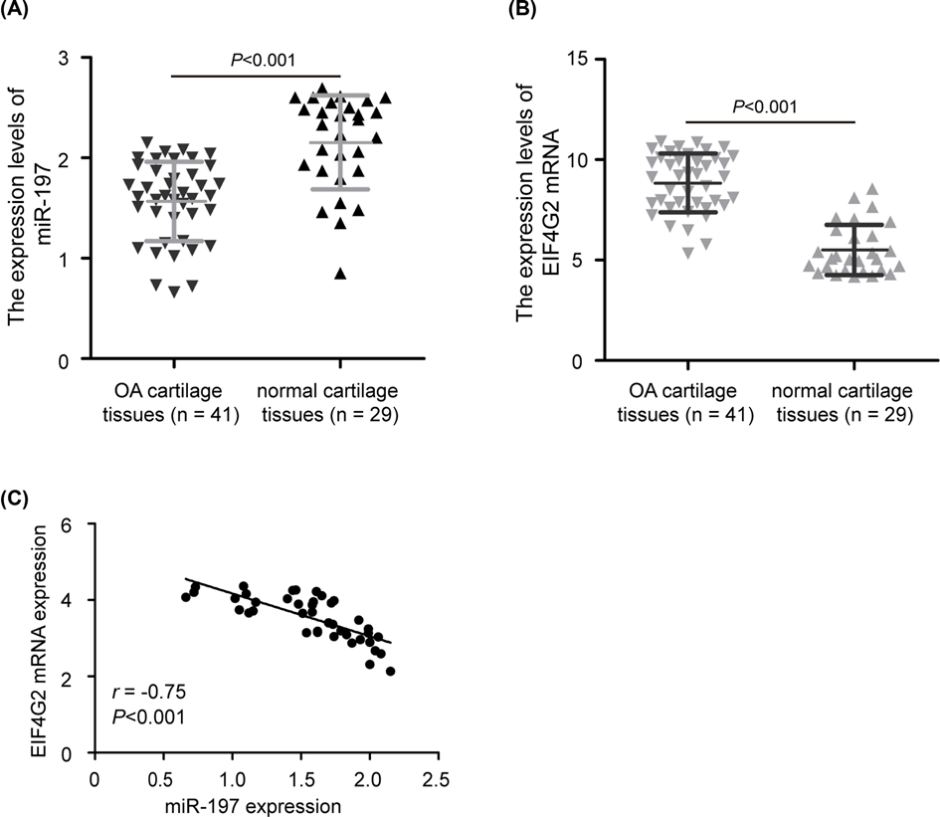
Expression of miR-197 and EIF4G2 in human osteoarthritis (OA) (**A**) Relative expression levels of miR-197 in osteoarthritis (OA) and normal cartilage tissues were detected by quantitative real-time PCR analysis. RNU6B was used as endogenous control of miR-197. (**B**) The relative expression of EIF4G2 mRNA was significantly up-regulated in OA cartilage tissues compared with normal cartilage tissues. GAPDH was used as endogenous control of EIF4G2 mRNA. (**C**) The inverse relationship was observed between miR-197 and EIF4G2 mRNA expression in OA cartilage tissues. The data were presented as the means ± standard deviation (SD) from three independent experiments in triplicate.

To determine whether miR-197 and EIF4G2 were dysregulated in OA patients, the expression levels of miR-197 and EIF4G2 in 41 OA cartilage tissues and 29 normal cartilage tissues were detected using quantitative real-time PCR analysis. As shown in Figure 1A, miR-197 expression was significantly decreased in OA cartilage tissues compared with normal cartilage tissues (*P*<0.001). However, EIF4G2 mRNA expression was increased in OA cartilage tissues compared with normal cartilage tissues (Figure 1B, *P*<0.001). We further analyzed the correlation between EIF4G2 and miR-197 expression in OA cartilage tissues by Spearman’s correlation analysis, and found that EIF4G2 mRNA expression was inversely correlated with miR-197 expression in OA cartilage tissues (Figure 1C, *r* = −0.75, *P*<0.001). These above data suggested that miR-197 and EIF4G2 may involved in OA-related pathogenesis.

### miR-197 regulates cell proliferation, migration, and inflammation in chondrocytes

**Figure 2:**
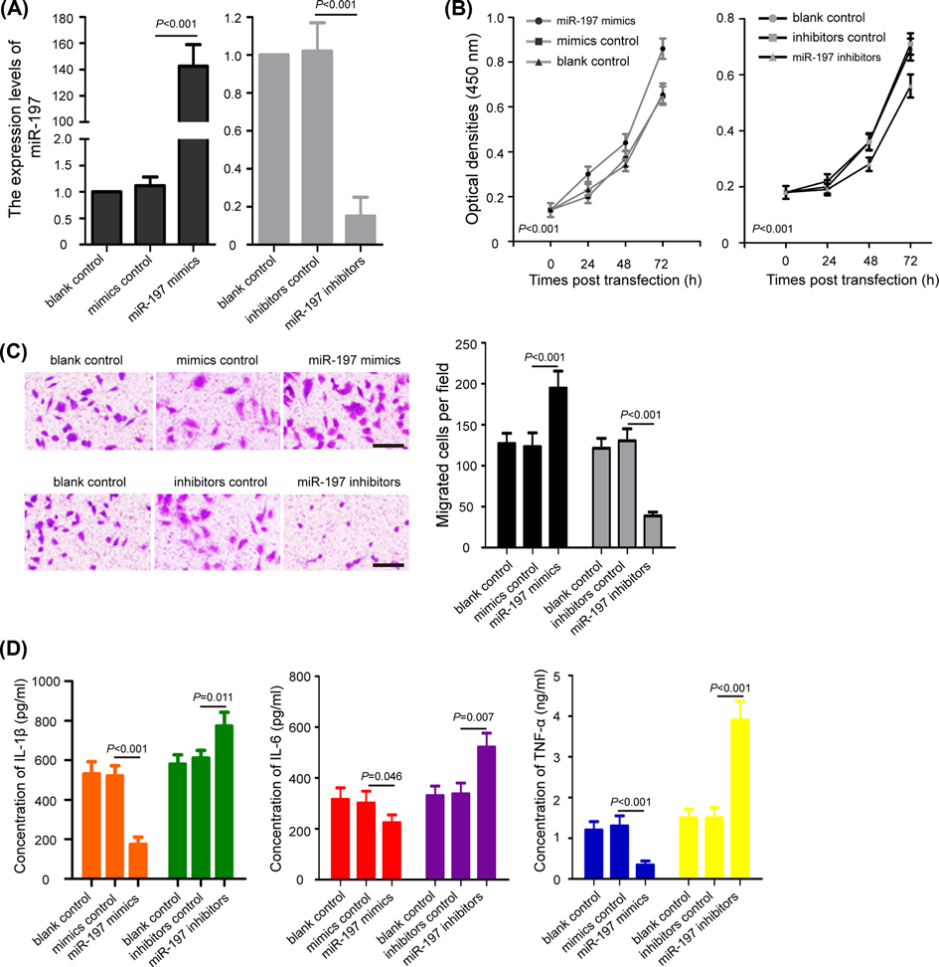
The effects of miR-197 on cell proliferation, migration, and inflammation in chondrocytes (**A**) Chondrocytes were transfected with miR-197 mimics, mimics control, miR-197 inhibitors, and inhibitors control for 48 h, and the expression levels of miR-197 were detected. (**B**) MTT assay reveal that overexpression of miR-197 promoted the proliferation of chondrocytes, while down-regulation of miR-197 suppressed chondrocytes growth. (**C**) Transwell assay showed that miR-197 mimics facilitated the migration of chondrocytes, whereas miR-197 inhibitors induced the opposite effects; scale bar: 100 μm. (**D**) ELISA assay analysis of the concentration of IL-1β, IL-6, and TNF-α in chondrocytes after miR-197 overexpression and knockdown. The data were presented as the means ± SD from three independent experiments in triplicate.

To investigate the potential roles of miR-197 on OA, primary human chondrocytes were isolated and transfected with miR-197 mimics, mimics control, miR-197 inhibitors, and inhibitors control. The efficiency of miR-197 overexpression and knockdown in chondrocytes was shown in Figure 2A (*P*<0.001). By using MTT assay, we found that overexpression of miR-197 clearly promoted the proliferation of chondrocytes, while down-regulation of miR-197 significantly suppressed chondrocytes growth (Figure 2B, *P*<0.001). Similarly, miR-197 mimics facilitated the migration of chondrocytes, while miR-197 inhibitors induced the opposite effects (Figure 2C, *P*<0.001). In addition, we analyzed the difference in cell inflammation after miR-197 overexpression and knockdown by ELISA assay. Results showed that chondrocytes transfected with miR-197 mimics showed a marked reduction in the expression of IL-1β, IL-6, and TNF-α compared with the mimics control groups (*P*<0.05), whereas miR-197 inhibitors significantly induced the expression of IL-1β, IL-6, and TNF-α compared with the inhibitors control groups (Figure 2D, *P*<0.05). These data suggested that miR-197 promotes proliferation and migration, but inhibits inflammation of chondrocytes.

### The expression of EIF4G2 was regulated by miR-197 in chondrocytes

**Figure 3:**
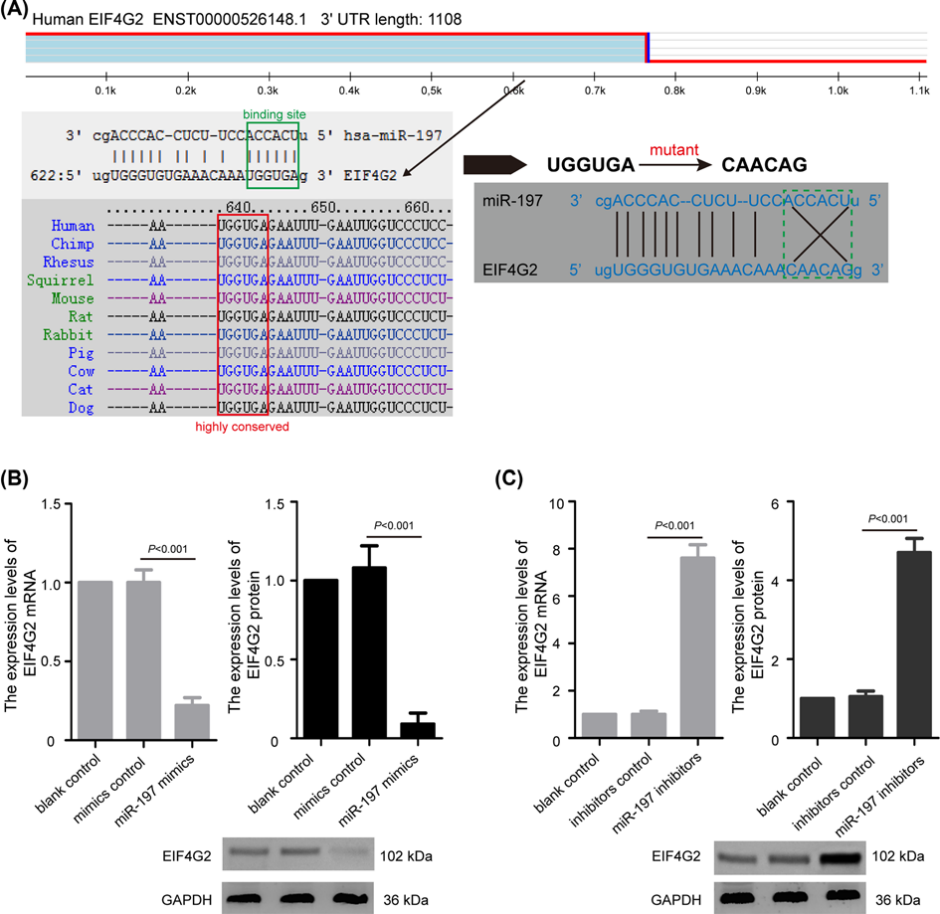
The expression of EIF4G2 is regulated by miR-197 in chondrocytes (**A**) Schematic diagram of the predicted targeting binding sites of miR-197 on the 3′-UTR sequences of EIF4G2 mRNA. (**B**) Quantitative real-time PCR analysis and Western blotting showed that overexpressed miR-197 significantly down-regulated EIF4G2 mRNA and protein expression in chondrocytes. (**C**) miR-197 suppression elevated EIF4G2 mRNA and protein expression in chondrocytes. The data were presented as the means ± SD from three independent experiments in triplicate.

To investigate the molecular mechanism of miR-197 in pathogenesis of OA, the potential target genes of miR-197 were predicted using TargetScanHuman 7.2 (http://www.targetscan.org/vert_72/), miRBase (http://www.mirbase.org/), and microRNA.org (http://www.microrna.org/microrna/). The EIF4G2 gene with high binding scores was selected from the overlapping gene set. The 3′-UTR sequences of EIF4G2 mRNA (UGGUGA) had a complementary binding site for miR-197 (ACCACU) (Figure 3A). Subsequently, we conducted quantitative real-time PCR analysis and Western blotting to detect the effects of miR-197 overexpression and knockdown on EIF4G2 expression in chondrocytes. In chondrocytes, overexpressed miR-197 significantly down-regulated EIF4G2 mRNA and protein expression in chondrocytes (Figure 3B, *P*<0.001), while miR-197 suppression strongly elevated EIF4G2 mRNA and protein expression (Figure 3C, *P*<0.001). The data demonstrated that miR-197 suppresses EIF4G2 expression in chondrocytes.

### EIF4G2 is a directly target gene of miR-197 in chondrocytes

**Figure 4:**
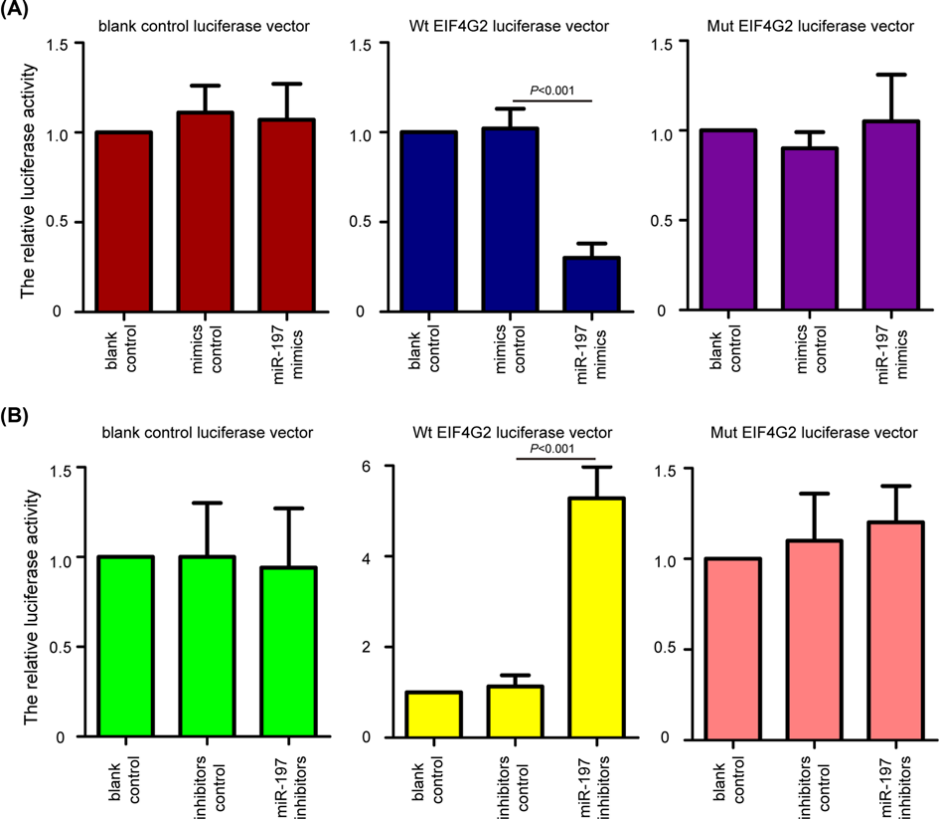
EIF4G2 is a directly target gene of miR-197 in chondrocytes (**A**) The chondrocytes were transfected with the Wt or Mut EIF4G2 luciferase vector in the presence of miR-197 overexpression. Luciferase reporter assay showed that miR-197 mimics significantly decreased the relative luciferase activity of Wt EIF4G2 luciferase vector, but not Mut EIF4G2 luciferase vector. (**B**) miR-197 inhibitors increased the relative luciferase activity of Wt EIF4G2 luciferase vector, but not Mut EIF4G2 luciferase vector. The data were presented as the means ± SD from three independent experiments in triplicate.

To confirm whether EIF4G2 was a direct target of miR-197 in chondrocytes, the luciferase reporter assay was performed. The chondrocytes were transfected with the Wt or Mut EIF4G2 luciferase vector in the presence of miR-197 overexpression and knockdown. The Wt EIF4G2 luciferase vector had a miR-197-binding sites, while the Mut EIF4G2 luciferase vector was lacked a miR-197 binding sites. It was observed that the overexpression of miR-197 decreased relative luciferase activity in the presence of the Wt EIF4G2 luciferase vector (Figure 4A, *P*<0.001), whereas the knockdown of miR-197 increased the relative luciferase activity of Wt EIF4G2 luciferase vector (Figure 4B, *P*<0.001). Similarly, no significant change in relative luciferase activity was observed in Mut EIF4G2 luciferase vector in the presence of miR-197 overexpression (Figure 4A) and knockdown (Figure 4B). The data suggested that EIF4G2 is a directly target gene of miR-197 in chondrocytes.

### miR-197 regulates proliferation, migration, and inflammation of chondrocytes through targeting EIF4G2

**Figure 5:**
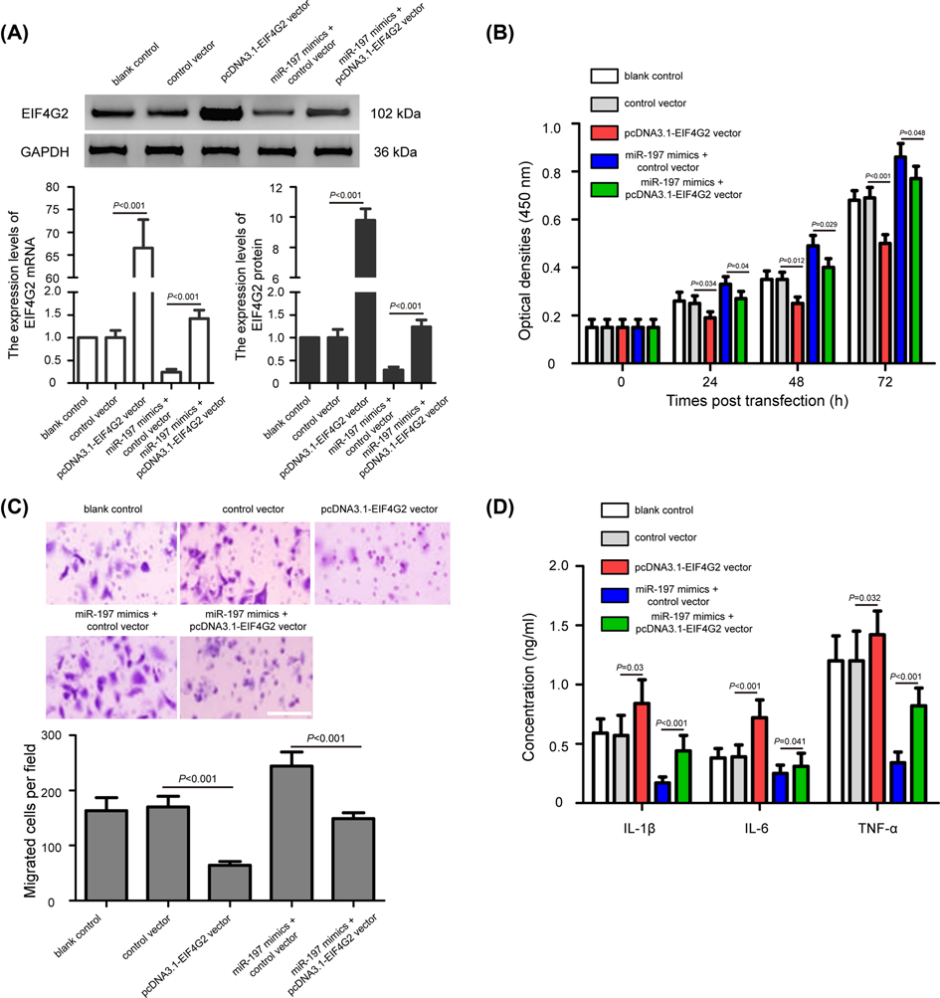
miR-197 regulates proliferation, migration, and inflammation of chondrocytes through targeting EIF4G2 (**A**) Relative expression of EIF4G2 mRNA and protein in miR-197 overexpressing chondrocytes transfected with pcDNA3.1-EIF4G2 vector and/or miR-197 mimics. (**B**) Up-regulation of cell proliferation induced by miR-197 overexpression was reversed by the pcDNA3.1-EIF4G2 vector. (**C**) The migration of chondrocytes induced by miR-197 overexpression could be reversed by EIF4G2 up-regulation; scale bar: 100 μm. (**D**) ELISA assay was conducted to analyze the expressions of IL-1β, IL-6, and TNF-α in miR-197 overexpressing chondrocytes after EIF4G2 up-regulation. The data were presented as the means ± SD from three independent experiments in triplicate.

**Figure 6:**
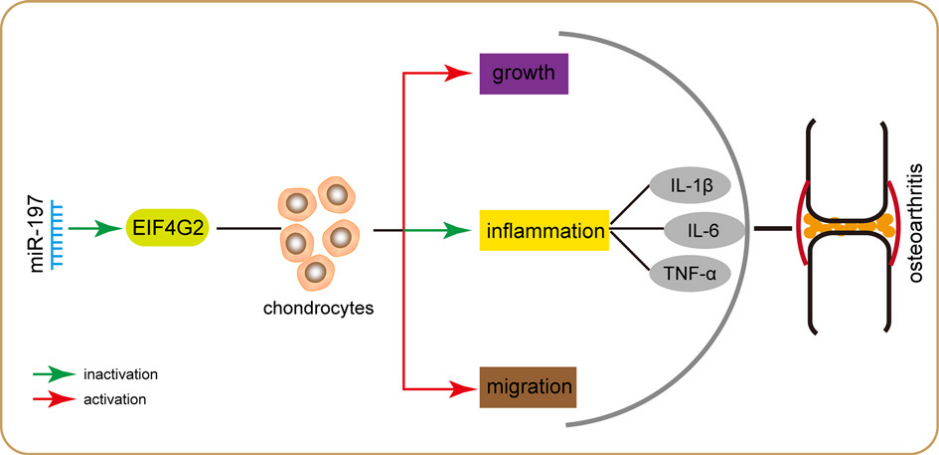
The mechanism graph of the function and regulatory mechanism of miR-197 in pathogenesis of OA miR-197 could promote proliferation and migration, and inhibit inflammation of chondrocytes by targeting EIF4G2.

To investigate whether EIF4G2 was involved in the regulation of miR-197 in pathogenesis of OA, we transfected the pcDNA3.1-EIF4G2 vector and control vector into miR-197 overexpressing chondrocytes. As shown in Figure 5A, pcDNA3.1-EIF4G2 vector could in strongly increase EIF4G2 mRNA and protein expression in chondrocytes compared with the control vector (*P*<0.001). Simultaneously, pcDNA3.1-EIF4G2 vector could increase the EIF4G2 expression in chondrocytes by miR-197 overexpression (*P*<0.001). Furthermore, MTT assay showed that the up-regulation of cell proliferation induced by miR-197 overexpression was reversed by the pcDNA3.1-EIF4G2 vector (Figure 5B, *P*<0.05). As shown in Figure 5C, a consistent result with findings in transwell assay was detected that the cell migration were markedly decreased in miR-197 overexpressing chondrocytes after EIF4G2 up-regulation (*P*<0.001). Besides, ELISA assay revealed that the inhibitory effects on IL-1β, IL-6, and TNF-α expression levels by miR-197 overexpression in chondrocytes could be partly abolished by the introduction of pcDNA3.1-EIF4G2 vector (Figure 5D, *P*<0.05). These results suggest that pcDNA3.1-EIF4G2 vector neutralized the effects of the miR-197 overexpression, indicating that miR-197 regulates proliferation, migration, and inflammation of chondrocytes through targeting EIF4G2. The mechanism graph of the function and regulatory mechanism of miR-197 in pathogenesis of OA is presented in Figure 6.

## Discussion

OA is a chronic degenerative osteochondral unit disease and begins with an aging related disruption of the cartilage tissues [1,2]. Since the underlying mechanism of OA is not fully understand, there is no specially therapy for OA treatment. Recently, increasing studies have demonstrated that the proliferation and inflammation of chondrocytes are closely associated with the pathogenesis and progression of OA [10]. The maintaining proliferation of chondrocytes is essential for maintaining the integrity of cartilage tissues. miRNAs are a class of non-coding molecules that regulate the expression of target genes and have been shown to participate in the biological regulation of chondrocytes [26]. For example, Lian et al. [27] showed that miR-128a represses chondrocyte autophagy and exacerbates knee OA by disrupting autophagy related 12 gene. Wang et al. [28] indicated that mR-590-5p targets transforming growth factor β1 to promote chondrocytes apoptosis and autophagy in response to mechanical pressure injury. Yu et al. [29] demonstrated that inhibition of miR-126 protects chondrocytes from IL-1β induced inflammation via up-regulation of Bcl-2. In the present study, we found that miR-197 was significantly decreased in OA cartilage tissues. miR-197 could promote cell proliferation and migration, and suppress inflammation of chondrocytes. We also found that EIF4G2 is a directly target gene of miR-197, and miR-197 executed its effects on chondrocytes through targeting EIF4G2.

Previous studies have shown that miR-197, a member of the miR-197 family, plays a vital role in the progression of multiple human cancers [19]. In cervical carcinogenesis, the expression of miR-197 was down-regulated in cancer tissues and cell lines, and overexpression of miR-197 inhibited growth and metastasis of cancer cells by directly targeting FOXM1 [30]. In hepatocellular carcinoma, miR-197-3p was down-regulated in carcinoma tissues and its expression correlated with aggressive clinicopathological characteristics [20]. miR-197 depletion exerts an tumor suppressive effect on the progression of gastric cancer [31]. Moreover, miR-197 has been shown to be suppressed cell proliferation, migration, and invasion in glioblastoma [32]. Hence, these studies demonstrated that miR-197 is involved in cell proliferation and migration. Nevertheless, the potential role of miR-197 involved in OA progression remains unknown. In this work, MTT and transwell assays were applied to determine the effects of miR-197 on the proliferation and migration abilities of chondrocytes. Our results showed that miR-197 overexpression promoted proliferation and migration. Conversely, miR-197 knockdown induced the opposite effects. The consistent results of cell proliferation and migration concluded that miR-197 might exert a protective effects on the pathogenesis of human OA.

The inflammation of articular cartilage and releases of inflammatory cytokines from chondrocytes play important roles in the degeneration of OA [33]. Studies have shown that miR-197 is important inflammatory inhibitor in human diseases [22]. For example, Estep et al. [34] found that miR-197 is significantly associated with pericellular fibrosis in non-alcoholic steatohepatitis patients, and levels of IL-6 in the serum negatively correlates with the expression levels of miR-197. Schulte et al. [35] demonstrated that serum-derived circulating miR-197 is identified as a predictor for cardiovascular death in a large patient cohort with coronary artery disease. Moreover, Cheng et al. [36] found miR-197 reverses hsa_circ_0068087 silence-induced HUVEC dysfunction and inflammation in the high glucose condition. However, no other data are available on the roles of miR-197 in the regulation of inflammatory responses associated with OA. In the present study, restoration of miR-197 significantly decreased inflammatory cytokines, including IL-1β, IL-6, and TNF-α expression, whereas knockdown of miR-197 led to a induction in these inflammatory mediators. These results suggested that miR-197 alleviates chondrocytes inflammatory injury of OA.

A large body of evidences have indicated that miRNAs reduce translation and/or degradation of target gene expression by targeting 3′-UTRs of their target mRNAs [12]. To investigate the mechanism of the effects of miR-197 on chondrocytes, further bioinformatics analyses was applied to predict the downstream target genes of miR-197. EIF4G2 was also found to act as a target of several miRNAs, including miR-379 in osteosarcoma [37], miR-139-5p in myeloid leukemia [38], and miR-520c-3p in diffuse large B-cell lymphoma [39]. Our study identified EIF4G2 as a potential target of miR-197. Here, we validated that expression of the EIF4G2 was elevated in human OA cartilage tissues, and found that EIF4G2 mRNA expression was inversely correlated with miR-197 expression in OA cartilage tissues. Consistent with these results, quantitative real-time PCR analysis and Western blotting revealed that overexpressed miR-197 significantly down-regulated EIF4G2 mRNA and protein expression in chondrocytes, while miR-197 suppression elevated EIF4G2 mRNA and protein expression. Significantly, luciferase reporter assay confirmed that miR-197 directly interacted with 3′-UTR sequences of EIF4G2 mRNA in chondrocytes. EIF4G2 is a vital mediator of translation initiation and plays an important role during cell mitosis [40]. Recently, Hu et al. [41] found EIF4G2 has a regulatory effects on cell viability, colony formation, and cell migration in chondrocyte CHON-001 cells. Furthermore, in order to explore whether miR-197 exerted its roles in chondrocytes by targeting EIF4G2. We transfected the pcDNA3.1-EIF4G2 vector into miR-197 overexpressing chondrocytes, and the results showed that EIF4G2 overexpression could reverse the effects of miR-197 mimics on chondrocytes proliferation, migration, and inflammation. Thus, these data suggested that EIF4G2 was the functional target gene of miR-197 in OA.

In conclusions, the present study demonstrated that miR-197 is down-regulated in OA cartilage tissues, while EIF4G2 is up-regulated. miR-197 promotes proliferation and migration, and inhibits inflammation of chondrocytes by targeting EIF4G2. Our results provide a basis for targeting the miR-197/EIF4G2 axis for the prevention and treatment of OA.

## Data Availability

The analyzed data sets generated during the study are available from the corresponding author on reasonable request.

## Abbreviations

ELISA: enzyme–linked immunosorbent assay
miRNA: microRNA
MTT: 3-(4,5-dimethylthiazol-2-yl)-2,5-di-phenyltetrazolium bromide
OA: osteoarthritis
IL: interleukin
TNF-a: tumor necrosis factor-a

## References

[1] Stewart H.L. and Kawcak C.E. (2018) The Importance of Subchondral Bone in the Pathophysiology of Osteoarthritis. Front. Vet. Sci. 5, 178 10.3389/fvets.2018.00178 30211173 PMC6122109

[2] Belluzzi E., Stocco E., Pozzuoli A., Granzotto M., Porzionato A., Vettor R. et al. (2019) Contribution of Infrapatellar Fat Pad and Synovial Membrane to Knee Osteoarthritis Pain. Biomed. Res. Int. 2019, 6390182 10.1155/2019/6390182 31049352 PMC6462341

[3] Poole A.R. (2012) Osteoarthritis as a whole joint disease. HSS J. 8, 4–6 10.1007/s11420-011-9248-6 23372516 PMC3295952

[4] Parkinson L., Waters D.L. and Franck L. (2017) Systematic review of the impact of osteoarthritis on health outcomes for comorbid disease in older people. Osteoarthr. Cartil. 25, 1751–1770 10.1016/j.joca.2017.07.008 28710026

[5] Loeser R.F., Collins J.A. and Diekman B.O. (2016) Ageing and the pathogenesis of osteoarthritis. Nat. Rev. Rheumatol. 12, 412–420 10.1038/nrrheum.2016.65 27192932 PMC4938009

[6] van den Bosch M.H.J. (2019) Inflammation in osteoarthritis: is it time to dampen the alarm(in) in this debilitating disease? Clin. Exp. Immunol. 195, 153–166 10.1111/cei.13237 30421798 PMC6330652

[7] Wang T. and He C. (2018) Pro-inflammatory cytokines: The link between obesity and osteoarthritis. Cytokine Growth Factor Rev. 44, 38–50 10.1016/j.cytogfr.2018.10.002 30340925

[8] Veronesi F., Della Bella E., Cepollaro S., Brogini S., Martini L. and Fini M. (2016) Novel therapeutic targets in osteoarthritis: Narrative review on knock-out genes involved in disease development in mouse animal models. Cytotherapy 18, 593–612 10.1016/j.jcyt.2016.02.001 27059198

[9] Evans C.H. (2018) Catering to chondrocytes. Sci. Transl. Med. 10, eaav7043 10.1126/scitranslmed.aav7043 30487250

[10] Zhang M. and Wang J. (2015) Epigenetics and Osteoarthritis. Genes Dis. 2, 69–75 10.1016/j.gendis.2014.12.005 25961070 PMC4421878

[11] Bartel D.P. (2004) MicroRNAs: genomics, biogenesis, mechanism, and function. Cell 116, 281–297 10.1016/S0092-8674(04)00045-5 14744438

[12] Bartel D.P. (2009) MicroRNAs: target recognition and regulatory functions. Cell 136, 215–233 10.1016/j.cell.2009.01.002 19167326 PMC3794896

[13] Schickel R., Boyerinas B., Park S.M. and Peter M.E. (2008) MicroRNAs: key players in the immune system, differentiation, tumorigenesis and cell death. Oncogene 27, 5959–5974 10.1038/onc.2008.274 18836476

[14] Zhang M., Lygrisse K. and Wang J. (2017) Role of MicroRNA in Osteoarthritis. J. Arthritis 6, 239 10.4172/2167-7921.1000239 28758052 PMC5533289

[15] Malemud C.J. (2018) MicroRNAs and Osteoarthritis. Cells 7, E92 10.3390/cells7080092 30071609 PMC6115911

[16] Yin C.M., Suen W.C., Lin S., Wu X.M., Li G. and Pan X.H. (2017) Dysregulation of both miR-140-3p and miR-140-5p in synovial fluid correlate with osteoarthritis severity. Bone Joint Res. 6, 612–618 10.1302/2046-3758.611.BJR-2017-0090.R1 29092816 PMC5717073

[17] Lu J., Ji M.L., Zhang X.J., Shi P.L., Wu H., Wang C. et al. (2017) MicroRNA-218-5p as a Potential Target for the Treatment of Human Osteoarthritis. Mol. Ther. 25, 2676–2688 10.1016/j.ymthe.2017.08.009 28919376 PMC5768591

[18] Duan Z.X., Huang P., Tu C., Liu Q., Li S.Q., Long Z.L. et al. (2019) MicroRNA-15a-5p Regulates the Development of Osteoarthritis by Targeting PTHrP in Chondrocytes. Biomed. Res. Int. 2019, 3904923 10.1155/2019/3904923 30949498 PMC6425345

[19] Wang D.D., Chen X., Yu D.D., Yang S.J., Shen H.Y., Sha H.H. et al. (2016) miR-197: A novel biomarker for cancers. Gene 591, 313–319 10.1016/j.gene.2016.06.035 27320730

[20] Ni J.S., Zheng H., Huang Z.P., Hong Y.G., Ou Y.L., Tao Y.P. et al. (2019) MicroRNA-197-3p acts as a prognostic marker and inhibits cell invasion in hepatocellular carcinoma. Oncol. Lett. 17, 2317–2327 30675297 10.3892/ol.2018.9848 PMC6341871

[21] Zhang N., Tian L., Miao Z. and Guo N. (2018) MicroRNA-197 induces epithelial-mesenchymal transition and invasion through the downregulation of HIPK2 in lung adenocarcinoma. J. Genet. 47, 47–53 10.1007/s12041-018-0881-4 29666324

[22] Cuppen B.V., Rossato M., Fritsch-Stork R.D., Concepcion A.N., Schenk Y., Bijlsma J.W. et al. (2016) Can baseline serum microRNAs predict response to TNF-alpha inhibitors in rheumatoid arthritis? Arthritis Res. Ther. 18, 189 10.1186/s13075-016-1085-z 27558398 PMC4997731

[23] Chen L., Li C., Peng Z., Zhao J., Gong G. and Tan D. (2013) miR-197 Expression in Peripheral Blood Mononuclear Cells from Hepatitis B Virus-Infected Patients. Gut Liver 7, 335–342 10.5009/gnl.2013.7.3.335 23710316 PMC3661967

[24] Naranda J., Gradisnik L., Gorenjak M., Vogrin M. and Maver U. (2017) Isolation and characterization of human articular chondrocytes from surgical waste after total knee arthroplasty (TKA). Peer J. 5, e3079 10.7717/peerj.3079 28344902 PMC5363257

[25] Schmittgen T.D. and Livak K.J. (2008) Analyzing real-time PCR data by the comparative C(T) method. Nat. Protoc. 3, 1101–1108 10.1038/nprot.2008.73 18546601

[26] D'Adamo S., Cetrullo S., Minguzzi M., Silvestri Y., Borzi R.M. and Flamigni F. (2017) MicroRNAs and Autophagy: Fine Players in the Control of Chondrocyte Homeostatic Activities in Osteoarthritis. Oxid. Med. Cell Longev. 2017, 3720128 28713485 10.1155/2017/3720128 PMC5497632

[27] Lian W.S., Ko J.Y., Wu R.W., Sun Y.C., Chen Y.S., Wu S.L. et al. (2018) MicroRNA-128a represses chondrocyte autophagy and exacerbates knee osteoarthritis by disrupting Atg12. Cell Death Dis. 9, 919 10.1038/s41419-018-0994-y 30206206 PMC6134128

[28] Wang J., Zhang Y., Song W., Ma T. and Wang K. (2018) microRNA-590-5p targets transforming growth factor beta1 to promote chondrocyte apoptosis and autophagy in response to mechanical pressure injury. J. Cell. Biochem. 119, 9931–9940 10.1002/jcb.27315 30117199

[29] Yu C.D., Miao W.H., Zhang Y.Y., Zou M.J. and Yan X.F. (2018) Inhibition of miR-126 protects chondrocytes from IL-1beta induced inflammation via upregulation of Bcl-2. Bone Joint Res. 7, 414–421 10.1302/2046-3758.76.BJR-2017-0138.R1 30034795 PMC6035362

[30] Hu Q., Du K., Mao X. and Ning S. (2018) miR-197 is downregulated in cervical carcinogenesis and suppresses cell proliferation and invasion through targeting forkhead box M1. Oncol. Lett. 15, 10063–10069 29928375 10.3892/ol.2018.8565 PMC6004723

[31] Liao Z., Li Y., Zhou Y., Huang Q. and Dong J. (2018) MicroRNA-197 inhibits gastric cancer progression by directly targeting metadherin. Mol. Med. Rep. 17, 602–611 29115517 10.3892/mmr.2017.7908

[32] Zhang G., Sun W., Zhu L., Feng Y., Wu L. and Li T. (2019) Overexpressed circ_0029426 in glioblastoma forecasts unfavorable prognosis and promotes cell progression by sponging miR-197. J. Cell. Biochem. 120, 10295–10302 10.1002/jcb.28313 30548670

[33] Berenbaum F., Griffin T.M. and Liu-Bryan R. (2017) Review: Metabolic Regulation of Inflammation in Osteoarthritis. Arthritis Rheumatol. 69, 9–21 10.1002/art.39842 27564539 PMC5341385

[34] Estep M., Armistead D., Hossain N., Elarainy H., Goodman Z., Baranova A. et al. (2010) Differential expression of miRNAs in the visceral adipose tissue of patients with non-alcoholic fatty liver disease. Aliment. Pharmacol. Ther. 32, 487–497 10.1111/j.1365-2036.2010.04366.x 20497147

[35] Schulte C., Molz S., Appelbaum S., Karakas M., Ojeda F., Lau D.M. et al. (2015) miRNA-197 and miRNA-223 Predict Cardiovascular Death in a Cohort of Patients with Symptomatic Coronary Artery Disease. PLoS ONE 10, e0145930 10.1371/journal.pone.0145930 26720041 PMC4699820

[36] Cheng J., Liu Q., Hu N., Zheng F., Zhang X., Ni Y. et al. (2019) Downregulation of hsa_circ_0068087 ameliorates TLR4/NF-kappaB/NLRP3 inflammasome-mediated inflammation and endothelial cell dysfunction in high glucose conditioned by sponging miR-197. Gene 709, 1–7 10.1016/j.gene.2019.05.012 31108165

[37] Xie X., Li Y.S., Xiao W.F., Deng Z.H., He H.B., Liu Q. et al. (2017) MicroRNA-379 inhibits the proliferation, migration and invasion of human osteosarcoma cells by targetting EIF4G2. Biosci. Rep. 37, BSR20160542 10.1042/BSR20160542 28381518 PMC5434889

[38] Emmrich S., Engeland F., El-Khatib M., Henke K., Obulkasim A., Schoning J. et al. (2016) miR-139-5p controls translation in myeloid leukemia through EIF4G2. Oncogene 35, 1822–1831 10.1038/onc.2015.247 26165837

[39] Mazan-Mamczarz K., Zhao X.F., Dai B., Steinhardt J.J., Peroutka R.J., Berk K.L. et al. (2014) Down-regulation of eIF4GII by miR-520c-3p represses diffuse large B cell lymphoma development. PLoS Genet. 10, e1004105 10.1371/journal.pgen.1004105 24497838 PMC3907297

[40] Coldwell M.J., Cowan J.L., Vlasak M., Mead A., Willett M., Perry L.S. et al. (2013) Phosphorylation of eIF4GII and 4E-BP1 in response to nocodazole treatment: a reappraisal of translation initiation during mitosis. Cell Cycle 12, 3615–3628 10.4161/cc.26588 24091728 PMC3903713

[41] Hu W., Zhang W., Li F., Guo F. and Chen A. (2016) miR-139 is up-regulated in osteoarthritis and inhibits chondrocyte proliferation and migration possibly via suppressing EIF4G2 and IGF1R. Biochem. Biophys. Res. Commun. 474, 296–302 10.1016/j.bbrc.2016.03.164 27105918

